# Editorial: Mechanobiology at multiple scales

**DOI:** 10.3389/fbioe.2023.1226198

**Published:** 2023-06-14

**Authors:** Yuhui Li, Guang-Kui Xu

**Affiliations:** ^1^ Université PSL, Commissariat à l'énergie atomique et aux énergies alternatives (CEA), Institut Pierre-Gilles De Gennes, CytoMorpho Lab, Paris, France; ^2^ Laboratory for Multiscale Mechanics and Medical Science, Department of Engineering Mechanics, SVL, School of Aerospace Engineering, Xi’an Jiaotong University, Xi’an, China

**Keywords:** mechanobiology, scaling, cells, proteins, organs

The significance of mechanical forces across multiple scales in biological systems cannot be understated. Understanding the impact of these forces on biological systems is essential for unraveling the complex interplay between mechanical stimuli and cellular responses. In addition, exploring the effects of mechanical forces at various scales provides valuable insights into either physiological or pathological conditions. Here, the aim of this Research Topic is dedicated to two points: 1) Highlighting the significance of mechanical forces across different scales of biological components. 2) Emphasizing the importance of recognizing the impact of mechanical forces not only in physiological conditions but also in pathological contexts, offering potential avenues for diagnostic and clinical advancements.

At the nanoscale, the interaction between receptors and ligands is influenced by mechanical forces, dictating cellular signaling and behavior. One typical example is the cell-cell communication process, representing a quintessential example of a phenomenon encompassing multiple receptors and ligands at the nanoscale that are anchored to adjacent membranes. These processes are mediated by the specific binding of receptors and ligands that are anchored to adjacent membranes. Li’s laboratory has made noteworthy progress in studying mechanotransduction in this domain, focusing on three aspects: 1) protein-membrane interactions, 2) single-molecule forces, and 3) bioelectrical microenvironments (An et al.). They reviewed recent progressive achievements in the field and concluded that the binding kinetics of proteins constitute a crucial parameter that guides intercellular and cell-cell communication. Furthermore, the influence of multi-scale coupling effects on binding affinity is expected to emerge as an important area of research in the future.

At the microscale level, mechanical forces play a critical role in cell mechanics and motility. For example, Yang’s laboratory has introduced an innovative experimental technique complemented by a corresponding computational fluid dynamics (CFD) model intended to investigate cell mechanics under dynamic loading conditions (Xu et al.). By applying Water-Hammer theories, they successfully evaluate and simulate the exerted stress on individual cells. This pioneering approach harmoniously merges microfluidic chips with a projectile pump, yielding insightful outcomes. This microfluidic-based methodology establishes a novel instrument for probing the dynamic mechanical properties of cells, presenting a fresh perspective for further exploration in this field. In another case, Zhou’s laboratory has presented a noteworthy investigation detailing an optical-tweezer-based platform that facilitates the assessment of both the chirality and frequency of human sperm rotation. These measurements can potentially serve as indicators of sperm motility and quality (Zhong et al.). To streamline the measurement system’s complexity, the research team opted to determine the orientation of the sperm head along the optical axis within the optical trap, utilizing the intensity distribution patterns of micron-sized particles positioned off-focus. This study not only unveils promising avenues for future research on sperm rolling but also underscores the significance of integrating artificial intelligence imaging analysis within intracytoplasmic sperm injection treatment.

Exploring the effects of mechanical forces at various scales provides valuable insights into either physiological or pathological conditions. In physiological contexts, understanding the mechanical cues that regulate cell behavior and tissue homeostasis can aid in designing biomaterials, tissue engineering strategies, and regenerative medicine approaches. On the other hand, in pathological conditions such as cancer, cardiovascular diseases, and musculoskeletal disorders, aberrant mechanical forces can drive disease progression and tissue dysfunction. Thus, comprehending the mechanobiological mechanisms across multiple scales holds great potential for developing diagnostics, therapeutics, and interventions targeting various diseases. The Research Topic also presents the related results from Qiao’s laboratory. They conducted a quantitative examination to explore the correlation between stent malapposition (SM) distance and stent thrombosis (Qu et al.). The findings reveal a notable rise in thrombus formation as the gap distance increases when the SM distance measures less than 150 μm. However, the thrombogenicity progression weakens once the gap distance exceeds 150 μm. Consequently, heightened attention is warranted when SM manifests with a 150 μm gap distance. Meanwhile, Long’s laboratory undertook a comprehensive review encompassing the hemodynamic changes induced by partial hepatectomy during liver regeneration and the decoupling of mechanical forces within hepatic sinusoids (Wu et al.). This exploration encompassed factors such as shear stress, mechanical stretch, blood pressure, and tissue stiffness. The study delved into potential mechanosensor and the associated signaling pathways. Additionally, Xu’s laboratory compiled a comprehensive summary elucidating the necessary biochemical signatures observed during cardiac fibrosis (Liu et al.). These signatures were classified into two distinct categories, static and dynamic, with a specific focus on the unique attributes of the heart. Researchers aim to pave the way for effective anti-fibrosis strategies in clinical therapy by comprehending these dynamic and static biomechanical characteristics.

In a separate investigation, Gong’s laboratory examined the biomechanical properties of articular cartilage and subchondral bone in guinea pigs with spontaneous hip osteoarthritis (Gao et al.). Their report highlights the occurrence of morphological degeneration in cartilage preceding the degeneration of mechanical properties. These findings provide novel insights into the structural and micromechanical interplay underlying hip osteoarthritis, serving as a theoretical foundation for comprehending its formation and progression. Furthermore, Panzetta’s laboratory expounded upon the effects of radiation on the expression ratio of YAP in the nucleus and cytoplasm, observing an increase in healthy cells and a decrease in breast cancer cells (La Verde et al.). These findings deepen our comprehension of the extracellular matrix’s role and shed light on the impact of X-rays on YAP and lamin A/C expression. Such insights can inform radiation therapy optimization through refined dosage and timing.

By investigating the mechanical forces at different scales, we can unravel the hierarchy and interconnectivity inherent to mechanobiology. This knowledge enables us to decipher the fundamental principles governing mechanotransduction, mechanoresponses, and mechanosensing in biological systems. Moreover, it provides a foundation for developing innovative technologies and methodologies that can detect, quantify, and manipulate mechanical forces at the microscale, nanoscale, and beyond. Recognizing the importance of mechanical forces across multiple scales is crucial for advancing our understanding of fundamental biological processes and their implications in health and disease. Integrating multidisciplinary approaches, including engineering, biology, and physics, can provide comprehensive insights into the mechanobiological complexities and pave the way for transformative advancements in various fields, ranging from regenerative medicine to drug discovery. While our Research Topic merely scratches the surface of the mechanobiology field, we anticipate these findings will contribute to the broader comprehension of the intricate dynamics involved when mechanical forces propagate within multi-scale biological systems.

